# Antiviral activity of ISG15 against classical swine fever virus replication in porcine alveolar macrophages via inhibition of autophagy by ISGylating BECN1

**DOI:** 10.1186/s13567-020-00753-5

**Published:** 2020-02-24

**Authors:** Cheng Li, Yifan Wang, Hongqing Zheng, Wang Dong, Huifang Lv, Jihui Lin, Kangkang Guo, Yanming Zhang

**Affiliations:** 1 College of Veterinary Medicine, Northwest A&F University, Yangling, Shaanxi China; 2 Tianjin Animal Husbandry and Veterinary Research Institute, Tianjin, China; 3 Henan University of Animal Husbandry and Economy, Zhengzhou, China; 4 School of Nursing, Southwest Medical University, Luzhou, Sichuan China

## Abstract

Interferons (IFNs) induce the expression of interferon-stimulated genes (ISGs) for defense against numerous viral infections, including classical swine fever virus (CSFV). However, the mechanisms underlying the effect of ISGs on CSFV infection are rarely reported. In this study, we demonstrate that IFN-α treatment induces upregulation of ISG15 and thus attenuates CSFV replication. To determine whether ISG15 is critical for controlling CSFV replication, we established porcine alveolar macrophages (PAMs) with stable overexpression or knockdown of ISG15. Overexpression of Flag-ISG15 significantly prevented CSFV replication, whereas loss of ISG15 led to abnormal proliferation of CSFV. Furthermore, upregulated ISG15 promoted beclin-1 (BECN1) ISGylation and dysfunction and subsequently inhibited autophagy, which is indispensable for CSFV replication. In addition, HECT and RLD domain containing E3 ubiquitin protein ligase 5 (HERC5), which functions to catalyze conjugation of ISG15 protein, was confirmed to interact with BECN1. Collectively, these results indicate that IFN-α restricts CSFV replication through ISG15-mediated BECN1 ISGylation and autophagy inhibition, providing insight into the mechanism of CSFV replication control by type I IFN. This mechanism may not be the only antiviral mechanism of ISG15; nonetheless, this study may contribute to the development of CSFV treatment and prevention strategies.

## Introduction

Classical swine fever (CSF), which is caused by the CSF virus (CSFV) of family *Flaviviridae*, is a highly contagious viral disease of pig and has serious socioeconomic implications [1]. CSFV is an enveloped RNA virus with positive-sense genomic RNA and an open reading frame that encodes a precursor polyprotein of 3898 amino acids that are co- and post-translationally processed into four structural proteins (C, E^rns^, E1, E2) and eight nonstructural proteins (N^pro^, p7, NS2, NS3, NS4A, NS4B, NS5A, NS5B) [2].

Type I interferon (IFN) has antiviral, antiproliferative, proapoptotic, and immunomodulatory activities. The production of type I IFN relies on the recognition of pathogen-associated molecular patterns by pattern recognition receptors, which induce an antiviral response by regulating synthesis of hundreds of proteins, inducing interferon-stimulated genes (ISGs) [3, 4]. RNA viruses of the family *Flaviviridae* are sensitive to type I IFN, and replication of CSFV is attenuated by treatment with IFN-α [5]. Although many studies have focused on screening and identifying antiviral ISGs and elucidating their antiviral mechanisms, only 2′,5′-oligoadenylate synthetase-like protein (OASL), myxovirus resistance protein 1 (Mx1), viperin, guanylate-binding protein 1 (GBP1), and IFN-inducible transmembrane proteins (IFITMs) have been identified to inhibit CSFV infection or replication [6–11].

Cells adapt to environmental changes through the initiation and execution of special processes, such as autophagy, which occurs in the event of poor survival conditions [12]. Autophagy processes include several stages, such as autophagy initiation signaling, autophagosome nucleation, autophagosome expansion, and autophagosome maturation [13]. It has been shown that CSFV infection triggers a complete autophagic response along with upregulation of autophagic markers, and induces formation of the membranes of autophagosome-like vesicles to promote viral replication in porcine kidney cells (PK-15) and porcine alveolar macrophages (PAMs) [14]. However, whether ISGs are involved in the regulation of autophagy during CSFV infection is largely unknown. Beclin-1 (BECN1), a highly conserved protein in mammals, is indispensable for canonical autophagy. BECN1 is a core component of the class III phosphatidylinositol 3-kinase (PI3KC3) complex, which is required for autophagosome nucleation and maturation. Furthermore, BECN1 regulates autophagic phosphatidylinositol 3-phosphate generation and recruits additional ATG proteins to orchestrate autophagosome formation [15].

ISG15 is one of the most abundantly induced ISGs and plays an important role in viral infection, but its role in regulating CSFV replication has not been extensively explored. ISG15 modifies its targets via a novel post-translational modification known as ISGylation, which spans a diverse array of biological processes and targets, including host and viral proteins [16]. Covalent conjugation of ISG15 to its target protein (ISGylation) is similar to ubiquitination and occurs through a sequential reaction catalyzed by UBE1L (E1)-activating, UbcH8 (E2)-conjugating, and HECT and RLD domain containing E3 ubiquitin protein ligase 5 (HERC5, E3) ligase enzymes [17]. ISGylation is a reversible process that requires ubiquitin-specific protease 18 (USP18) to remove ISG15 from the target protein [18]. Herein, we investigated the expression of ISG15 and ISGylation during CSFV infection. Experiments with shUSP18, ISG15 overexpression, and ISG15 mutant cells confirmed that the anti-CSFV action of ISG15 was ISGylation-dependent. BECN1 was ISGylated through ISG15 during CSFV infection, leading to autophagy-deficient CSFV reduction. Finally, BECN1 was demonstrated to interact with ISGylation E3 ligase.

## Materials and methods

### Cells, medium, and virus

The PAM cell line 3D4/2 [American Type Culture Collection (ATCC), Manassas, VA, USA; CRL-2845] was cultured in RPMI 1640 medium (Gibco, Grand Island, NY, USA) with 100 mL/L (10%) fetal bovine serum (Gibco). Human embryonic kidney (HEK-293T; ATCC) cells were cultured in Dulbecco’s minimal essential medium (DMEM, Gibco) with 10% fetal bovine serum (FBS, Gibco). IFN-α was purchased from Novoprotein (Shanghai, China), and 3-methyladenine (3MA) was purchased from Sigma-Aldrich (St. Louis, MO, USA). Rapamycin was obtained from Cell Signaling Technology (Boston, MA, USA). The Shimen strain of CSFV was purchased from the Control Institute of Veterinary Bio-products and Pharmaceuticals of China. All experiments related to CSFV were conducted in a P3 biosafety laboratory and strictly performed according to the Laboratory Biosafety Manual in the laboratory.

### Real-time quantitative polymerase chain reaction (RT-qPCR)

The relative mRNA expression of ISGs and CSFV was detected by RT-qPCR. Primer pairs are listed in Table 1. Total RNA from PAMs was isolated using TRIzol (Thermo Fisher Scientific, Waltham, MA, USA) and reverse transcribed into cDNA using PrimeScript RT reagent kit (Vazyme, Nanjing, China). RT-qPCR was performed using Ultra SYBR Mixture (CWBIO, Beijing, China) according to the manufacturer’s protocol. Relative fold changes in gene expression were normalized against *β*-actin expression using the 2^−ΔΔ*C*t^ threshold method.

**Table 1:** **Primers used for RT-qPCR**

Primer	Sequence (5′–3′)	Usage
ISG15-F	AGGGAACTGAAGGTGAAGATG	RT-qPCR for detection of ISG15
ISG15-R	CAGACGCTGCTGGAAGG	
MX1-F	TCTGTAAGCAGGAGACCATCAACT	RT-qPCR for detection of MX1
MX1-R	TTTCTCGCCACGTCCACTATC	
β-actin-F	CAAGGACCTCTACGCCAACAC	RT-qPCR for detection of β-actin
β-actin-R	TGGAGGCGCGATGATCTT	
CSFV-F	GAGAAGGACAGCAGAACTAAGC	RT-qPCR for detection of CSFV
CSFV-R	TTACCGCCCATGCCAATAGG	

### Western blot

The protein samples of interest were separated by 12% sodium dodecyl sulfate-polyacrylamide gel electrophoresis (SDS-PAGE) and transferred to polyvinylidene difluoride membranes (Millipore, Burlington, MA, USA). Following blocking in 5% skim milk at room temperature for 2 h, the membranes were immersed in the prepared primary antibody, which included mouse anti-β-actin monoclonal antibody (MAb) (Cell Signaling Technology), mouse anti-Myc MAb (Cell Signaling Technology), mouse anti-FLAG MAb (Sigma), mouse anti-ISG15 MAb (Cusabio, Wuhan, China), rabbit anti-LC3-II polyclonal antibody (PAb) (Sigma), and mouse anti-BECN1 MAb (Sigma), and incubated at 4 °C overnight with shaking. After washing with Tris-buffered saline containing 0.5% Tween 20 (TBST), the membranes were incubated with horseradish peroxidase (HRP)-conjugated secondary antibodies at room temperature for 2 h, followed by three washes in TBST. The protein bands were analyzed using an image analysis system (Bio-Rad) after incubation with enhanced chemiluminescence (ECL) reagent. β-actin served as an internal control.

### Construction and transfection of plasmid

Based on the genetic sequences of porcine *ISG15*, *USP18*, *HERC5*, and *BECN1* (GenBank: EU647216.1, NM_213826.1, XM_021100766.1, and NM_001044530.1), PCR primers for amplification were generated (Table 2). *ISG15* was inserted into the lentivirus plasmid pCDH-CMV-MCS-EF1 (CMV) with a Flag-tag to generate CMV-ISG15 and was cloned into pcDNA3.1 with a Flag-tag to generate Flag-ISG15 (3.1-ISG15GG). Synthesis of 3.1-ISG15AA from 3.1-ISG15GG was performed by directed mutagenesis with a Phusion site-directed mutagenesis kit (Thermo Fisher Scientific) following the manufacturer’s instructions. *BECN1* was cloned into pcDNA3.1 or pGEX-6p-1 to create Flag- or glutathione S-transferase (GST)-tagged BECN1, respectively. The N-terminus of *BECN1* fused with the C-terminal sequence LRLRGG of *ISG15* was inserted into pcDNA3.1 to generate Flag-tagged ISG15-BECN1. *HERC5* was amplified by PCR and inserted into pcDNA3.1 or pGEX-6p-1 to generate Myc- or GST-tagged HERC5. As for the short hairpin RNA (shRNA) plasmid, three pairs of shRNA for *ISG15* or *USP18*, as well as scrambled control shRNA (shN), were designed using the RNAi Designer website. All shRNAs were inserted into pCDH-U6-GreenPuro. The shRNA sequences are listed in Table 3. CMV, pcDNA3.1, and pCDH-U6-GreenPuro plasmids and pGAG, pREV, and pVSV-G helper plasmids were conserved in our laboratory. 3.1-Flag-RFP (RFP cloned into pcDNA3.1 with a Flag-tag) was constructed and conserved in our laboratory. To transiently transfect the plasmids into PAMs, a mixture containing 4 μg plasmids, 6 μL Turbofect (Thermo Fisher Scientific), and 400 μL Opti-MEM was added to the PAMs and incubated for 8 h. Then, the medium was refreshed with 10% FBS 1640 and incubated for another 24 h for subsequent experiments.

**Table 2:** **Primers used for plasmid construction**

Primer	Sequence (5′–3′)	Usage
CMV-ISG15-F	CGGAATTCATGGGTAGGGAACTGAAGGTGAAGA	Amplification of ISG15
CMV-ISG15-R	CGGGATCCGCACTCGGTGGGGTGCTCCC	
3.1-ISG15AA-A2	TGGCCCTGTCCCCGCCGCCCGCAGGCGCAG	Amplification of ISG15 mutant
3.1-ISG15AA-B1	CTGCGCCTGCGGGCGGCGGGGACAGGGCCA	
Flag-ISG15-F	CGGAATTCTATGGGTAGGGAACTGAAGGTGAAGA	Amplification of ISG15
Flag-ISG15-R	CGGGATCCGCACTCGGTGGGGTGCTCC	
Myc-BECN1-F	CGGAATTCTATGGAGGGGTCTAAGACATCCA	Amplification of BECN1
Myc-BECN1-R	CGGGATCCTTTGTTATAAAACTGTGAGGATACCC	
Flag-BECN1-F	CGGAATTCCATGGAGGGGTCTAAGACATCCA	Amplification of BECN1
Flag-BECN1-R	CGGGATCCTTTGTTATAAAACTGTGAGGATACCC	
Flag-ISG15-BECN1-A2	CTACAGAATCTGGGGAGGTAGCCCCCCCGCAGGCGCAGATT	Amplification of ISG15-BECN1
Flag-ISG15-BECN1-B1	ATCTGCGCCTGCGGGGGGGCATGGAGGGGTCTAAGACATCCA	
Myc-HERC5-F	TGC TCTAGA ATGGAGCGGAGACCACGGAG	Amplification of HERC5
Myc-HERC5-R	CG GGATCC GCCAAATCCCCTGTGGTTGC	
GST-BECN1-F	CGGAATTCATGGAGGGGTCTAAGACATCCA	Amplification of BECN1
GST-BECN1-R	CGGGATCCTTTGTTATAAAACTGTGAGGATACCC	
GST-HERC5-F	CG GGATCC ATGGAGCGGAGACCACGGAG	Amplification of HERC5
GST-HERC5-R	CCG CTCGAG TCAGCCAAATCCCCTGTGGTT	

Underlines show mutagenesis sites.

**Table 3:** **Short hairpin RNA (shRNA) sequences**

shRNA	Sequence (5′–3′)
shISG15-1-F	GATCCGACCAGTTCTGGCTGACTTTCCAAGAGGAAAGTCAGCCAGAACTGGTCTTTTTG
shISG15-1-R	AATTCAAAAAGACCAGTTCTGGCTGACTTTCCTCTTGGAAAGTCAGCCAGAACTGGTCG
shISG15-2-F	GATCCGCCTATGTGCACCGTGTATATCAAGAGATATACACGGTGCACATAGGCTTTTTG
shISG15-2-R	AATTCAAAAAGCCTATGTGCACCGTGTATATCTCTTGATATACACGGTGCACATAGGCG
shISG15-3-F	GATCCGTGCACCGTGTATATGAATCTCAAGAGAGATTCATATACACGGTGCACTTTTTG
shISG15-3-R	AATTCAAAAAGTGCACCGTGTATATGAATCTCTCTTGAGATTCATATACACGGTGCACG
shUSP18-1-F	GATCCGGTCGGTTTGCACAACATTGGCAAGAGCCAATGTTGTGCAAACCGACCTTTTTG
shUSP18-1-R	AATTCAAAAAGGTCGGTTTGCACAACATTGGCTCTTGCCAATGTTGTGCAAACCGACCG
shUSP18-2-F	GATCCGCCTTAACTCCCTGATTCAGGCAAGAGCCTGAATCAGGGAGTTAAGGCTTTTTG
shUSP18-2-R	AATTCAAAAAGCCTTAACTCCCTGATTCAGGCTCTTGCCTGAATCAGGGAGTTAAGGCG
shUSP18-3-F	GATCCGCCTACTGTCTCCAGAAGTACCAAGAGGTACTTCTGGAGACAGTAGGCTTTTTG
shUSP18-3-R	AATTCAAAAAGCCTACTGTCTCCAGAAGTACCTCTTGGTACTTCTGGAGACAGTAGGCG

Underlines show loop ring.

### Cell viability assay

The cell viability assay was performed with the Cell Counting Kit-8 (CCK-8) (Beyotime) according to the manufacturer’s instructions.

### Establishment and detection of ISG-overexpression and -knockdown cell lines

CMV plasmid containing *ISG15* or pCDH-U6 plasmids carrying shISG15 or shUSP18 were co-transfected into HEK-293T cells with three ancillary plasmids (pGAG, pREV, and pVSV-G) by Turbofect. These cells were cultured in DMEM with 2% FBS for 16 h, and the medium was replaced with DMEM containing 10% FBS, 0.01 mM cholesterol (Sigma), 0.01 mM l-α-phosphatidylcholine (Sigma), 1:1000 diluted Chemically Defined Lipid (Invitrogen, Carlsbad, CA, USA), and 4.0 mM l-glutamine (Invitrogen) followed by another 48 h of incubation. The supernatants were collected and centrifuged at 1500 *g* to obtain the 5 types of lentiviruses (CMV, CMV-ISG15, shN, shISG15, and shUSP18). Five lentiviruses were added to PAMs in a 6-well culture plate, respectively. The medium was exchanged with fresh medium at 8 h after infection of lentivirus, followed by incubation for another 48 h. CMV and shN served as negative controls. Puromycin (Thermo Fisher Scientific) was used to screen positive cells in complete growth medium at a final concentration of 2 μg/mL. To observe the screened positive cells that exhibited green fluorescence, a fluorescence inversion microscope (Nikon, Tokyo, Japan) was used. Western blot was performed to confirm the overexpression or knockdown of ISGs.

### Virus titration by immunofluorescence assay (IFA)

IFAs were performed to estimate the virus titers of CSFV in the cellular supernatant. PAMs were inoculated with cellular supernatant in a 96-well culture plate with 10 groups generated by a tenfold dilution series (10^−1^–10^−10^) for 48 h, and eight repetitions for each dilution were performed. A negative control was generated by culture with medium without CSFV. These cell samples were fixed by 1:1 fixing liquid (acetone:methanol) at −20 °C for 20 min and permeabilized by 0.1% Triton X-100 in a 4 °C refrigerator for 20 min. Permeabilized cells were incubated with 5% skim milk for 2 h at room temperature, followed by incubation with positive CSFV serum conserved in our laboratory at 4 °C for about 16 h. After washing, cells were incubated with fluorescein isothiocyanate (FITC)-conjugated rabbit anti-pig IgG antibody (Sigma) for 2 h. Between each step of the procedure, cells were washed three times using phosphate-buffered saline (PBS) or PBS with Tween 20 in the case of antibody washing. Negative controls were used for background subtraction. FITC-positive cells were observed and counted under a fluorescence inversion microscope (Nikon). The number of positive and negative wells of each dilution was recorded. The viral titers were expressed as 50% tissue culture infectious dose (TCID_50_)/mL.

### Confocal immunofluorescence microscopy

To analyze the effect of different treatment or transfection on autophagosomes, accumulation of LC3 puncta was quantified. Cells in 35-mm culture dishes were fixed by 1:1 fixing liquid (acetone:methanol) at 4 °C for 30 min following three washes with PBS for 10 min each. Then, the cell samples were permeabilized by 0.1% Triton X-100 at 4 °C for 20 min and blocked with 5% skim milk at room temperature for 2 h. Cells were incubated with a rabbit anti-LC3 PAb (Sigma) as a primary antibody and Alexa Fluor 488 AffiniPure goat anti-rabbit IgG (H + L) antibody (Yeasen, Shanghai, China) as a secondary antibody. Nuclei were counterstained with 4′,6-diamidino-2-phenylindole at room temperature for 10 min and imaged by laser scanning confocal microscopy (LSM510 META, Zeiss, Oberkochen, Germany). In most cases, > 12 cells were analyzed for each set, and each experiment was repeated three times. The average amount of LC3 puncta per cell from each group was analyzed with Image J and is presented graphically.

### Transmission electron microscopy (TEM)

PAMs were seeded in a 100-mm culture dish and treated with IFN-α, rapamycin, or CSFV, while the mock group was incubated with an equivalent amount of 1640 medium. After 48 h, the cells were collected in a 1.5-mL Eppendorf tube, centrifuged at 1000 *g* at 4 °C for 10 min, and then immobilized by 2.5% glutaraldehyde (Solarbio, Bejing, China). Fixed cells were sliced and observed with TEM.

### Co-immunoprecipitation (Co-IP) and GST pull-down assays

Flag-BECN1 and Myc-HERC5 plasmids were constructed for Co-IP assay, and irrelevant Flag-RFP served as a negative control. PAMs seeded in a 6-well culture plate were co-transfected with 2 μg of Flag-BECN1 and Myc-HERC5 using Turbofect and cultured for 48 h. After incubation, PAMs were collected and lysed by immunoprecipitation (IP) lysis buffer containing phenylmethanesulfonyl fluoride (Beyotime). The negative control was subjected to the same procedure. The protein supernatant was collected through centrifugation at 12 000 *g* at 4 °C and used for Co-IP assay with Anti-Flag M2 Affinity Gel (Sigma) or Anti-c-Myc Agarose Affinity Gel (Sigma) in accordance with the manufacturer’s instructions. After three washes with TBS, the immunoprecipitate was added to SDS-PAGE loading buffer and denatured for Western blot utilizing anti-Flag or anti-Myc antibodies.

To explore the direct interaction between BECN1 and HERC5, GST-BECN1 and GST were expressed in *Escherichia coli* BL21 cells, and Myc-HERC5 was expressed in HEK-293T cells. The GST pull-down tests were performed using the Pierce GST Protein Interaction Pull-Down Kit (Thermo Fisher Scientific) based on the manufacturer’s instructions. Glutathione agarose resin slurry was added to each of the two Pierce spin columns, after which a mixture of 200 μL TBS and 200 μL pull-down lysate blend was added. The columns were centrifuged for 45 s at 1500 *g* four times to balance the glutathione agarose resin. GST and GST-BECN1 were incubated with two of the balanced glutathione agarose resin samples at 4 °C for 60 min, followed by centrifugation and washing. Then, Myc-HERC5 was injected into the mixture and incubated at 4 °C for 2 h. Finally, elution buffer containing 3.1 mg GST per milliliter of TBS was used to separate the precipitate, which was centrifuged and boiled with SDS-PAGE loading buffer for 5 min. The interaction between BECN1 and HERC5 in the samples was detected by Western blot assay with anti-Myc antibody. To further confirm the interaction, GST-HERC5 and Flag-BECN1 were evaluated with the same method, and the eluted proteins were detected by Western blot assay with anti-Flag antibody.

### Statistical analysis

Data are presented as the mean ± standard deviation (SD) of three independent experiments. Student’s *t*-test was used for all statistical analyses. Differences in each group were considered to be significant with *P* values less than 0.05.

## Results

### CSFV replication is restricted by ISG15 in PAMs

**Figure 1:**
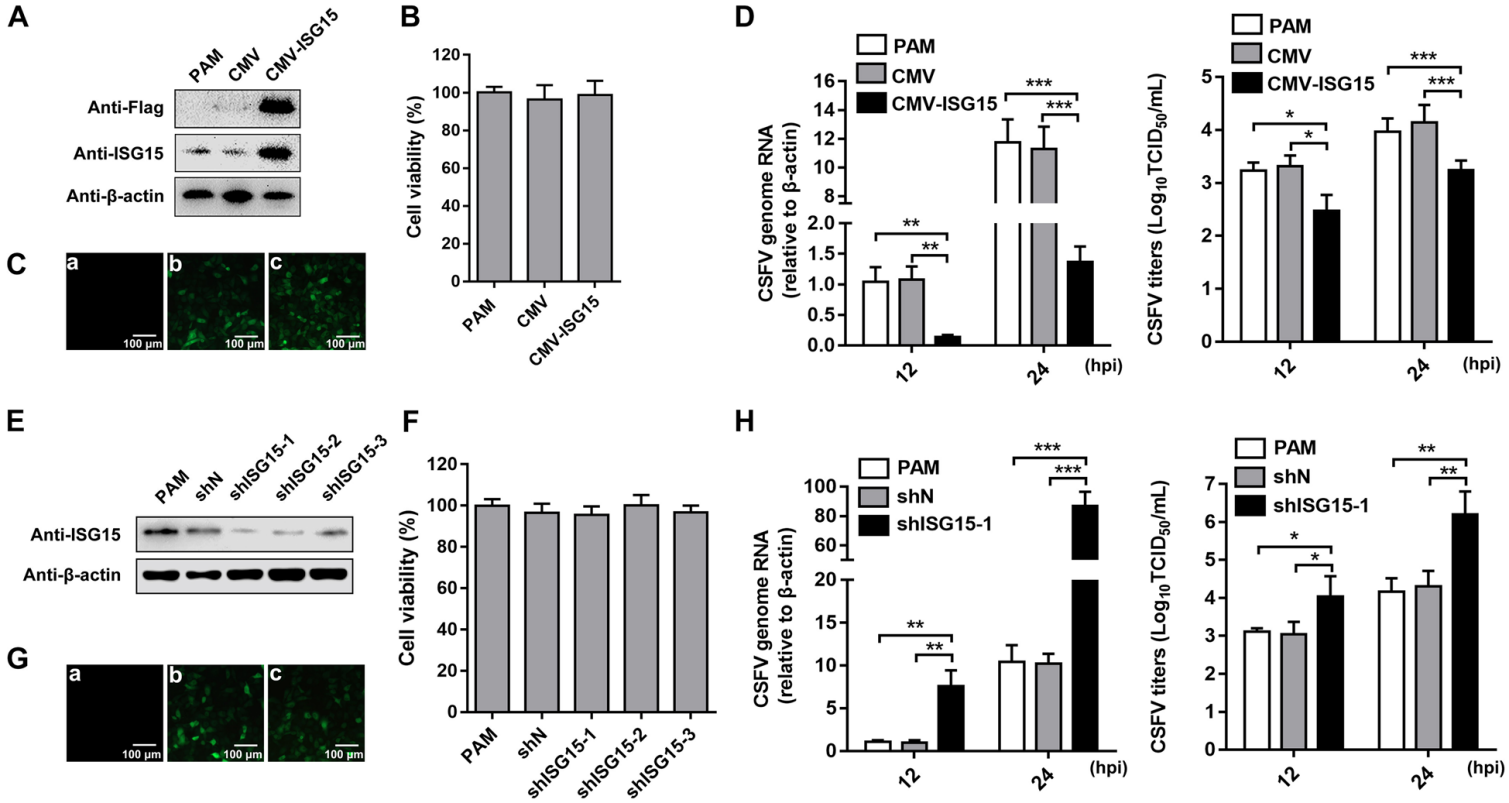
**CSFV replication is restricted by ISG15 in PAMs.** PAMs stably overexpressing ISG15 were constructed by lentiviral transduction. **A** Expression of ISG15 protein in PAMs and, CMV and CMV-ISG15 cells was determined by Western blot using an anti-Flag and an anti-ISG15 antibody, respectively. β-actin served as an internal control. **B** Cell viability of cell lines stably overexpressing ISG15. **C** GFP reporter expression was detected on mock-infected cells (a), CMV-infected cells (b) and l CMV-ISG15-infected cells (c). Scale bars, 100 μm. CSFV genomic RNA and viral titers **D** were analyzed in CMV and CMV-ISG15 cells at 12 and 24 hpi (MOI = 1) by RT-qPCR and IFA, respectively. **E** Expression of ISG15 protein was probed by Western blot. **F** Cell viability of cell lines with stable knockdown of ISG15. **G** GFP reporter expression was detected in mock-infected PAMs (a), shN-infected cells (b) and shISG15-1-infected cells (c). Scale bars, 100 μm. ShN and shISG15 cells were incubated with CSFV (MOI = 1). **G** CSFV genomic RNA was quantified in shN and shISG15 cells at 12 and 24 hpi. **H** The extracellular viral titers were quantified and expressed as TCID_50_/mL. Data (**B**, **D**, **F**, and **H**) represent the mean ± SD of three independent experiments and were measured in technical duplicate. Comparisons between groups were calculated using Student’s *t*-test. *P < 0.05; **P < 0.01; ***P < 0.001

To determine the role of ISG15 in CSFV infection, we constructed a cell line stably overexpressing Flag-ISG15 protein (CMV-ISG15 cells) by lentivirus. PAMs stably transfected with an empty vector CMV by lentivirus was regarded as a negative control (CMV cells). Overexpression of Flag-ISG15 in PAMs was easily detected by anti-Flag or anti-ISG15 antibody (Figure 1A) and did not affect cell proliferation and viability (Figure 1B). Western blot results showed that the efficiency of transfection was high (with anti-Flag antibody) and the exogenous expression of ISG15 was successful (with anti-ISG15 antibody). The expression of GFP indicated the efficiency of transfection. Green fluorescence was visualized in CMV and CMV-ISG15 cells under an inverted fluorescence microscope, but no green fluorescence was detected in the mock-transfected cells (Figure 1C). The positively transfected cell rate was approximately 100%. Compared with the transcriptional levels of CSFV in PAMs or CMV cells, those in CMV-ISG15 cells were significantly inhibited at 12 hours post-infection (hpi) and 24 hpi, respectively. CSFV titers in culture supernatants of CMV-ISG15 cells significantly decreased compared with those in CMV cells at 12 hpi and 24 hpi (Figure 1D). These data suggested that overexpression of Flag-ISG15 inhibits CSFV replication in PAMs.

To explore the effect of endogenous ISG15 on CSFV propagation, three PAM cell lines stably tranfected with shISG15-1, shISG15-2 and shISG15-3 and a negative control (shN cells) mediated by lentivirus infection were constructed. ISG15 expression in shISG15-1 cells was decreased compared with that in shN cells, exhibiting the highest knockdown efficiency (Figure 1E), and did not affect cell proliferation and viability (Figure 1F). The expression of GFP was used to indicate the transfection efficiency. Green fluorescence was visualized in shN and shISG15-1 cells under an inverted fluorescence microscope (Figure 1G). The rate of positively transfected cells was approximately 100%. Thus, shISG15-1 cells, referred to as shISG15 cells hereafter, were used for subsequent experiments. PAMs and, shISG15 and shN cells were infected with CSFV (multiplicity of infection [MOI] = 1), and CSFV genomic RNA levels and the progeny virus titers were dramatically increased in shISG15 cells compared with those in PAMs or shN cells at both 12 hpi and 24 hpi (Figure 1H). These results demonstrated that ISG15 is essential for controlling CSFV propagation in PAMs.

### ISG15 is induced by IFN-α and contributes to IFN antiviral activity against CSFV

**Figure 2:**
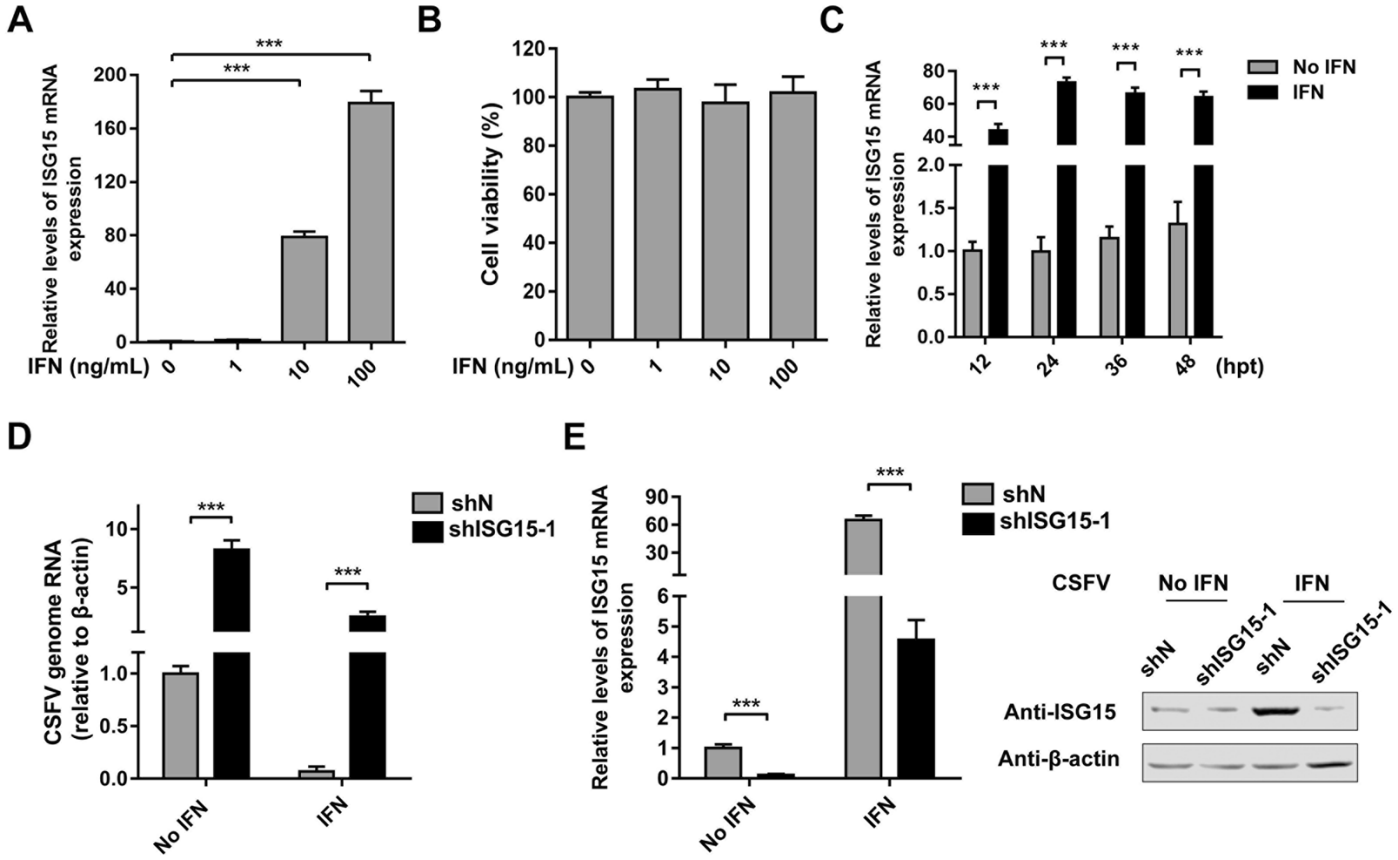
**ISG15 is induced by IFN-α and contributes to IFN antiviral activity against CSFV.** PAMs were treated with different amounts of IFN-α (0, 1, 10, and 100 ng/mL) for 24 h. **A***ISG15* expression was determined by RT-qPCR. **B** Cell viability was examined by MTT assay. **C** Mock- and IFN-α-treated (10 ng/mL) PAMs were harvested at the indicated times post-treatment. *ISG15* expression was quantified by RT-qPCR. IFN-α (10 ng/mL) was added as a positive control. ShISG15 and shN cells were mock-treated or treated with IFN-α (10 ng/mL) for 24 h prior to CSFV infection (MOI = 1). **D** CSFV genomic RNA was quantified by RT-qPCR. **E** ISG15 mRNA and protein levels were analyzed by RT-qPCR and Western blot. Data (**A**, **B**, **C**, **D**, and **E**) represent the mean ± SD of three independent experiments and were measured in technical duplicate. Comparisons between groups were calculated using Student’s *t*-test. ***P < 0.001

To confirm that ISG15 can be induced by type I IFN in PAMs, the cells were treated with different amounts of IFN-α. As expected, the expression of *ISG15* was rapidly and robustly induced by IFN-α in a dose-dependent manner, whereas the cell viability displayed no considerable changes (Figures 2A and B). Furthermore, *ISG15* expression was highly induced at 12 h (43.24-fold), 36 h (56.14-fold), and 48 h (52.38-fold) and peaked at 24 h (69.99-fold) (Figure 2C). ShISG15 and shN cells were pretreated with IFN-α for 24 h as a positive control, followed by CSFV (MOI = 1) infection. As shown in Figures 2D and E, IFN-α pretreatment significantly increased ISG15 expression and inhibited CSFV replication in shN cells, whereas knockdown of ISG15 resulted in a lower response to IFN-α treatment and aberrant CSFV replication, indicating that ISG15 is indispensable for the IFN-α-mediated antiviral effect.

### ISG15 expression is upregulated in CSFV-infected PAMs

**Figure 3:**
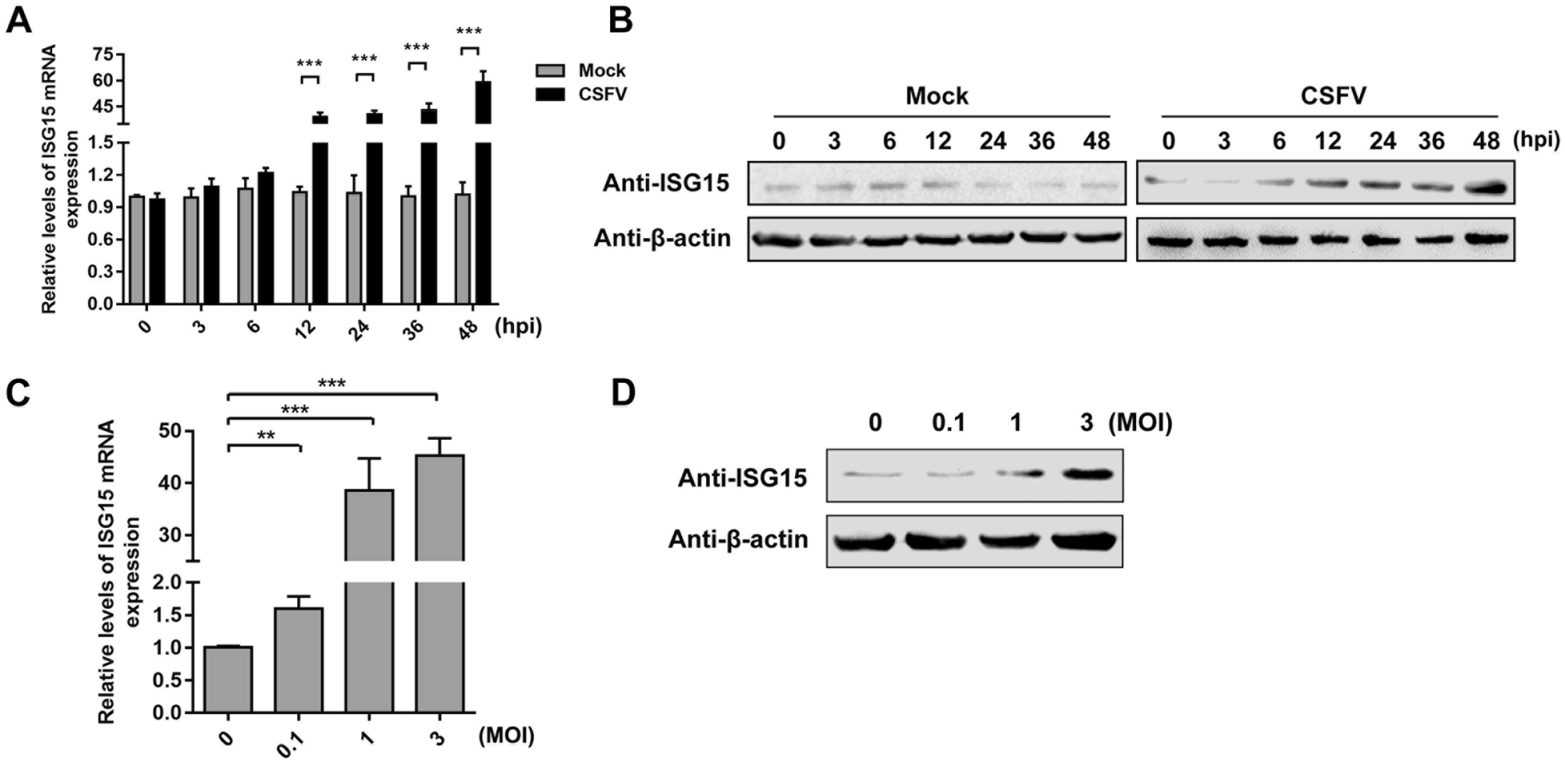
**ISG15 expression is upregulated in CSFV-infected PAMs.A**, **B** Expression of ISG15 in PAMs incubated with CSFV (MOI = 1) at 0, 3, 6, 12, 24, 36, and 48 hpi. **A***ISG15* expression was quantified by RT-qPCR. and Western blot. **C**, **D** Expression of ISG15 in PAMs incubated with CSFV (MOI = 0.1, 1, and 3). The expression of ISG15 was quantified by RT-qPCR (**C**) and Western blot (**D**) at 24 hpi. β-actin served as an internal control. Data (**A**, **C**) represent the mean ± SD of three independent experiments and were measured in technical duplicate. Comparisons between groups were calculated using Student’s *t*-test. **P < 0.01; ***P < 0.001

ISG15 expression of PAMs infected with CSFV (MOI = 1) was analyzed at the indicated times post-infection. The results showed that ISG15 expression was significantly upregulated at 12, 24, 36, and 48 hpi compared with that in the mock PAMs at both the mRNA and protein levels (Figures 3A, B). Intriguingly, following infection with different doses of CSFV (MOI = 0.1, 1, or 3) for 24 h, both mRNA and protein levels of ISG15 were upregulated (Figures 3C, D).

### ISGylation is involved in the antiviral activity of ISG15 against CSFV

**Figure 4:**
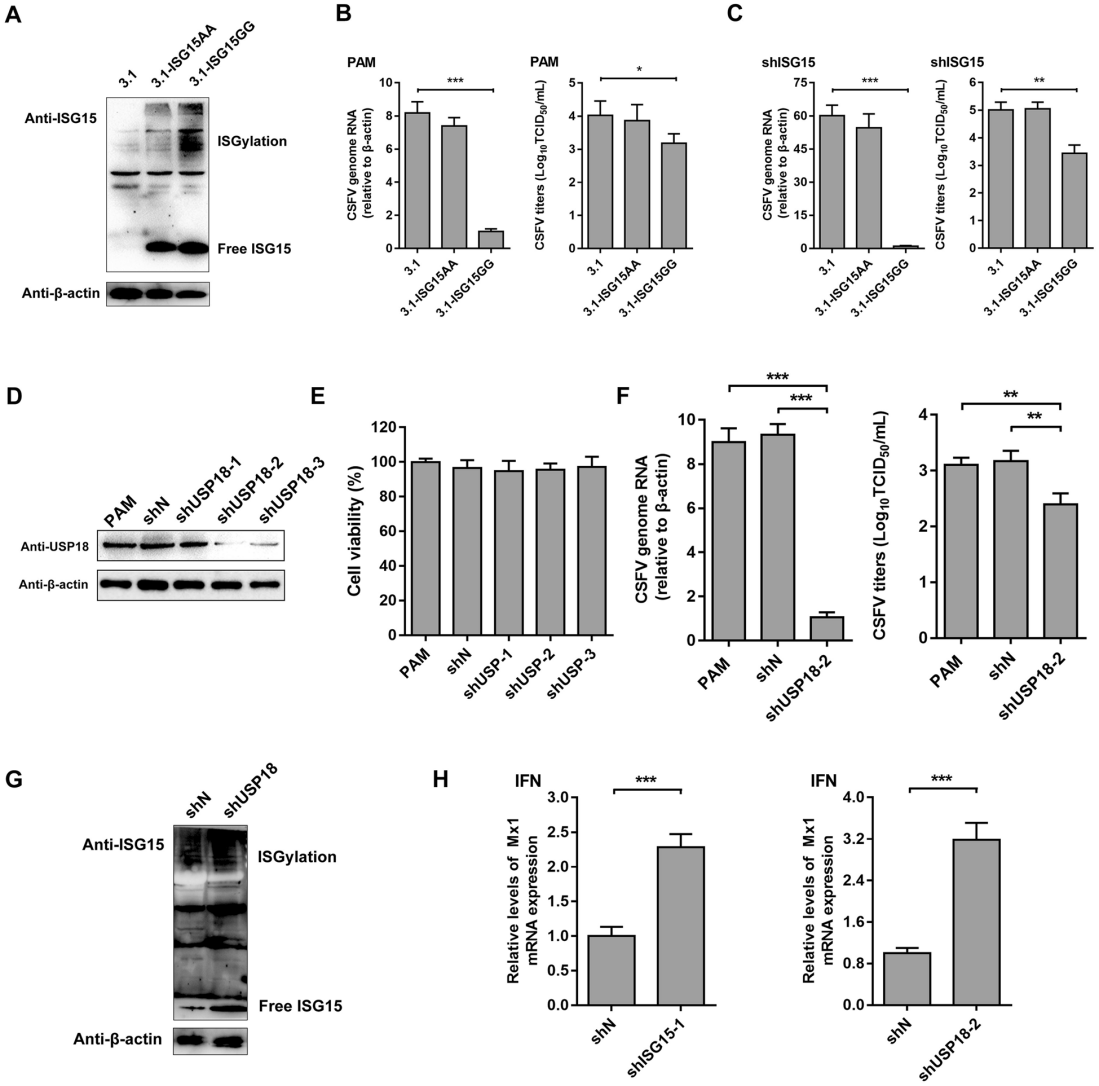
**ISGylation is involved in the antiviral activity of ISG15 against CSFV.A** Expression of ISGylated protein was detected by Western blot with an anti-ISG15 antibody. **B**, **C** Effects of ISGylation on CSFV replication. PAMs **B** and shISG15 cells **C** were transfected with 3.1, 3.1-ISG15AA, and 3.1-ISG15GG plasmid followed by CSFV infection for another 24 h (MOI = 1). CSFV replication was quantified by RT-qPCR and IFA, respectively. **D**, **E** PAMs with stable knockdown of USP18 were constructed by lentiviral transduction. **D** Expression of USP18 protein was detected by Western blot. **E** Cell viability with stable knockdown of USP18. ShN and shUSP18 cells were pretreated with IFN-α and then infected with CSFV (MOI = 1). **F** CSFV replication was quantified by RT-qPCR and IFA, respectively. **G** Expression of ISGylated protein was detected by Western blot with an anti-ISG15 antibody. **H***Mx1* expression was analyzed by RT-qPCR. Data (**B**, **C**, **E**, **F**, and **H**) represent the mean ± SD of three independent experiments and were measured in technical duplicate. Comparisons between groups were calculated using Student’s *t*-test. **P < 0.01; ***P < 0.001

To determine whether ISG15 exerts its anti-CSFV activity in a conjugated manner, a mutated form of *ISG15* (3.1-ISG15AA) was constructed via substituting GG with AA by site-directed mutagenesis. In 3.1-ISG15AA-transfected cells, only the free ISG15 level increased compared with that in the control, whereas the ISGylation level did not increase as much as that in 3.1-ISG15GG-transfected cells, suggesting that 3.1-ISG15AA-transfected cells lost the ability to conjugate with the target protein (Figure 4A). PAMs transfected with 3.1 were used as a control. CSFV genomic RNA levels and viral titers in 3.1-ISG15GG-transfected PAMs were significantly decreased compared with those in 3.1-transfected PAMs (Figure 4B). To exclude the influence of basal levels of ISG15, similar experiments were performed in shISG15 cells, which yielded similar results (Figure 4C). These results revealed that CSFV can be inhibited by conjugative ISG15. Furthermore, USP18, an ISG15-specific deconjugating protease that cleaves conjugation of ISG15, was knocked down in PAMs. We constructed cell lines stably transfected with USP18 shRNAs (shUSP18-1, shUSP18-2 and shUSP18-3) and a negative control (shN cells). The knockdown efficiency of shUSP18-2 was the highest at the protein level, and the viability of these cells was similar to that of mock-transduced PAMs which served as a control (Figures 4D, E). As shown in Figures 4F and G, compared with levels in the shN cells, knockdown of USP18 led to an upregulation of ISG15 conjugates and decrease in CSFV replication in IFN-pretreated cells, indicating that USP18 deficiency promoted ISGylation to inhibit CSFV replication.

Because both ISG15 and USP18 regulate the type I IFN signaling pathway, we evaluated *Mx1* (another ISG) expression in ISG15-knockdown cells and USP18-knockdown cells following IFN-α pretreatment and subsequent infection with CSFV. As shown in Figure 4H, knockdown of ISG15 expression upregulated *Mx1* expression, and a similar result was obtained in USP18-knockdown cells. However, knockdown of ISG15 and knockdown of USP18 showed opposite effects on CSFV replication (Figures 2H and 3H), suggesting that the effect of ISGylation was more pronounced than the effect of the IFN pathway in terms of inhibition of CSFV. These results suggested that ISG15 can impair CSFV replication in an ISGylation-dependent manner.

### ISG15 inhibits autophagy, which is required for CSFV replication

**Figure 5:**
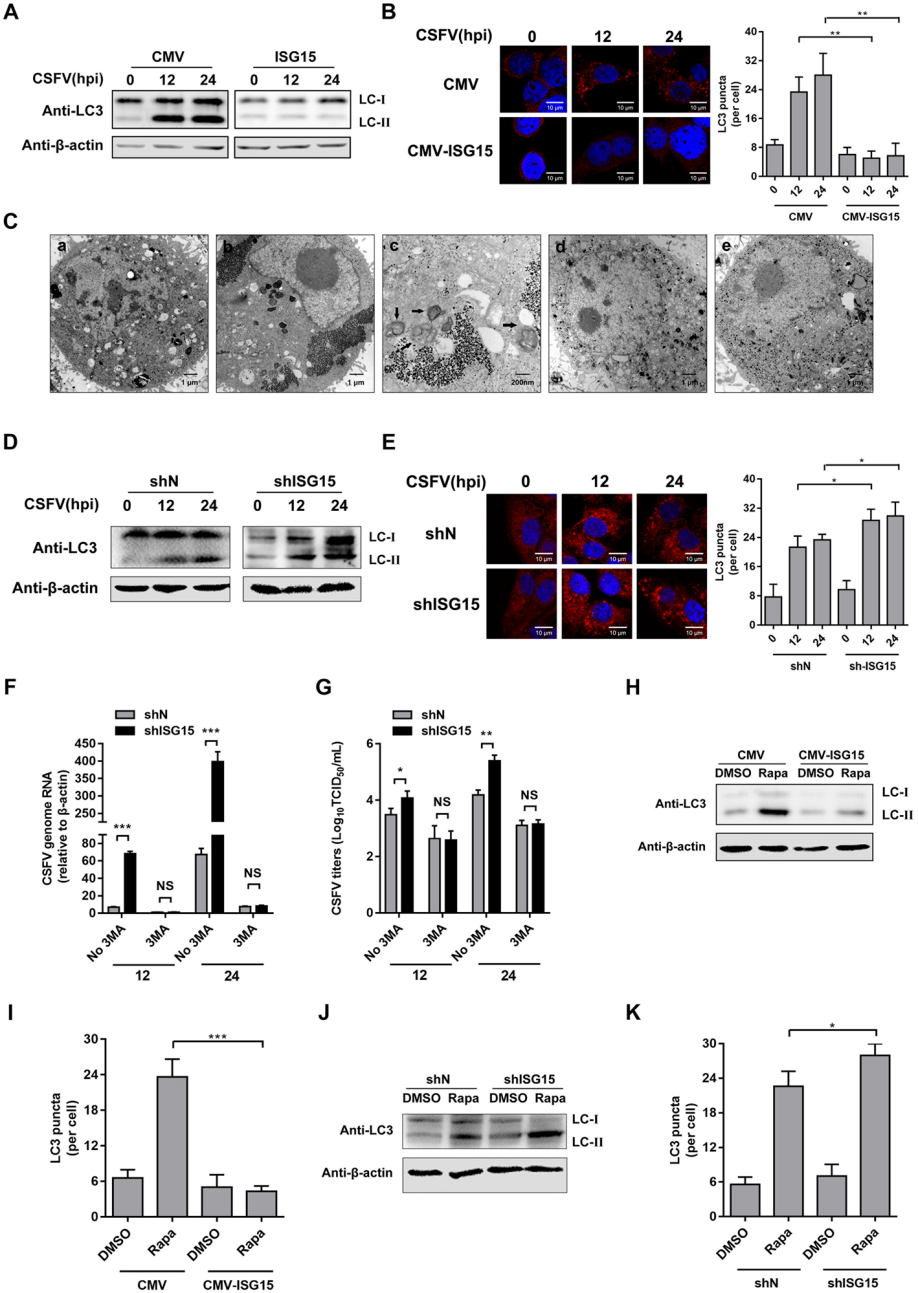
**ISG15 inhibits autophagy, which is required for CSFV replication.** CMV, CMV-ISG15, shN, and shISG15 cells were infected with CSFV (MOI = 1) for 12 h and 24 h. LC3 protein (**A**, **D**) was analyzed by Western blot, and LC3 puncta (**B**, **E**) in PAMs were observed by confocal immunofluorescence microscopy. Cells were fixed and subjected to IFA by immunostaining with anti-LC3 antibody. Representative cells are shown. Scale bar = 10 μm. The number of LC3 puncta per cell was determined by image analysis. **C** Transmission electron microscopy images. CMV and CMV-ISG15 cells were mock-infected or infected with CSFV (MOI = 1) for 24 h and studied by electron microscopy. Representative images of CMV cells (a), CSFV-infected CMV cells (b), magnified view of autophagosome-like vesicles (c), CMV-ISG15 cells (d), and CSFV-infected CMV-ISG15 cells (e) are shown. Scale bars = 1 μm (a, b, d, and e) and 200 nm (c). Black arrows indicate the structures with the characteristics of autophagosomes. **F**, **G** ShN and shISG15 cells were pretreated with 3MA for 4 h after 1 h of virus adsorption, after which the cells were provided medium with 3MA. After 24 h, CSFV replication was quantified by RT-qPCR and IFA, respectively. CMV and CMV-ISG15 cells (H and I) or shN and shISG15 cells (**J**, **K**) were treated with DMSO or rapamycin. Expression of LC3 protein (**H**, **J**) and LC3 puncta (**I**, **K**) in PAMs were quantified by image analysis. Data (**B**, **E**, **F**, **G**, **I**, and **K**) represent the mean ± SD of three independent experiments and were measured in technical duplicate. Comparisons between groups were calculated using Student’s *t*-test. *P < 0.05; **P < 0.01; ***P < 0.001

To test whether ISG15 interferes with CSFV-induced autophagy, protein expression of LC3 and the number of fluorescent puncta of LC3 were assessed in ISG15-overexpressing and ISG15-knockdown cells at various times post-infection with CSFV. LC3 is a widely used marker in autophagy research; during autophagy induction, LC-I is converted to LC3-II. As shown in Figures 5A and B, protein levels of LC3-II and accumulation of LC3 puncta were significantly reduced in CMV-ISG15 cells at both 12 hpi and 24 hpi. Similarly, the number of single- or double-membrane vesicles in the cytoplasm of CMV cells was higher than that in CMV-ISG15 cells (Figure 5C). As expected, knockdown of ISG15 obviously up-regulated both LC3-II expression and the fluorescent signal (Figures 5D and E). 3MA has been shown to inhibit autophagy via inhibition of autophagosome formation in PAMs during CSFV infection [19]. To further confirm that the antiviral action of ISG15 is mediated by direct inhibition of autophagy during CSFV infection, shN and shISG15 cells were treated with 3MA and infected with CSFV. As expected, knockdown of ISG15 expression failed to promote CSFV replication in the presence of 3MA (Figure 5F, G). These results demonstrated that ISG15 inhibits viral-induced autophagy to control CSFV replication. To determine whether ISG15 could also inhibit nonviral-induced autophagy, ISG15-overexpressing and ISG15-knockdown cells were treated with rapamycin, an autophagy inducer. The results showed that overexpression of ISG15 downregulated rapamycin-induced autophagy (Figures 5H and I). In shISG15 cells, rapamycin induced a more obvious increase in LC3-II protein and LC3 puncta accumulation than in shN cells (Figures 5J and K). Taken together, these results suggested that ISG15 can inhibit the autophagy process induced by both CSFV and rapamycin.

### Autophagy protein BECN1 is ISGylated by ISG15

**Figure 6:**
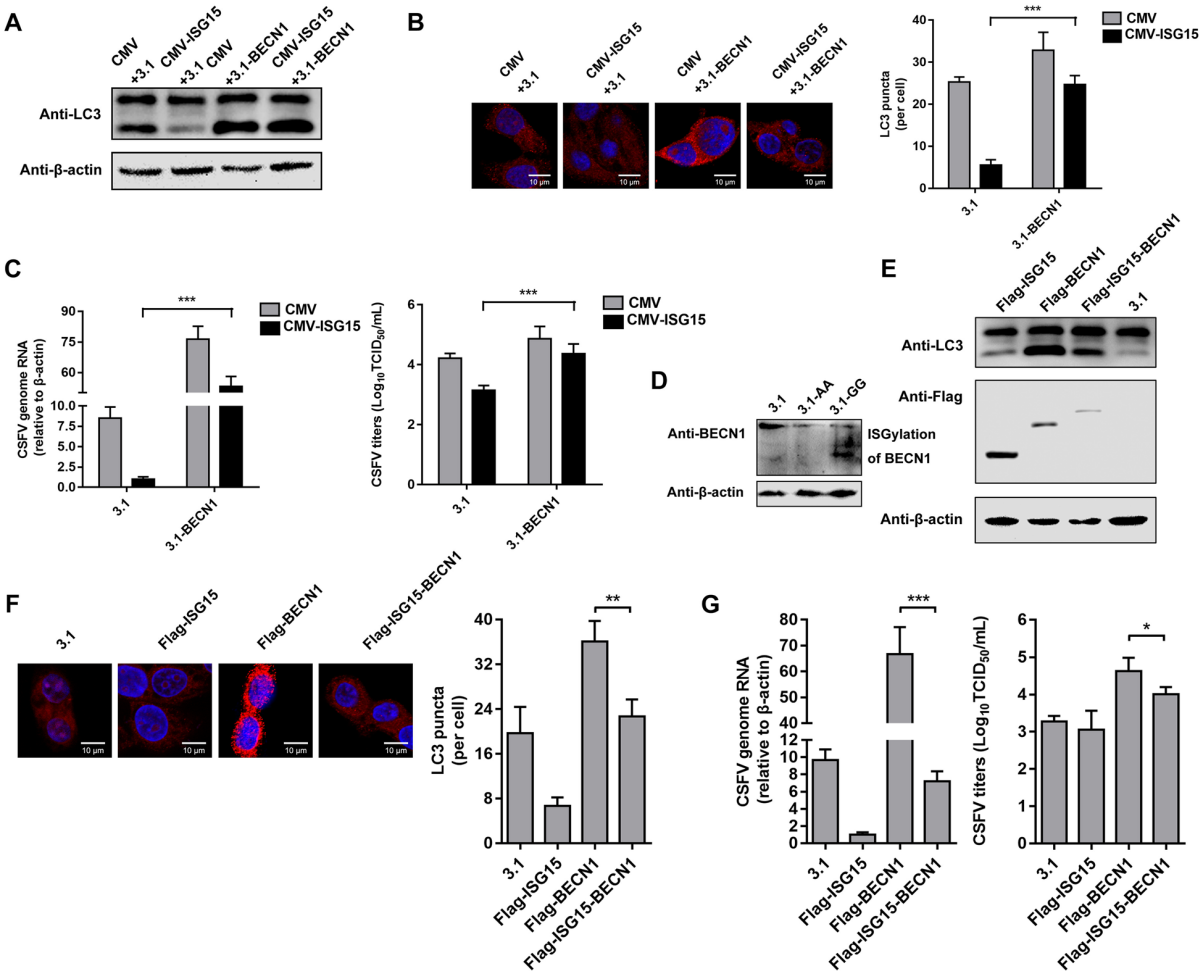
**Autophagy protein BECN1 is ISGylated by ISG15.A**–**C** Influence of ISG15 on autophagy and CSFV replication in BECN1-overexpressing cells. Expression of LC3 protein (**A**) and the number of LC3 puncta per cell (**B**) were determined by Western blot and image analysis, respectively. **C** CSFV replication was quantified by RT-qPCR and IFA, respectively. **D** ISGylation of BECN1 was detected in cells transfected with 3.1, 3.1-ISG15AA, and 3.1-ISG15GG plasmids. PAMs were transfected with 3.1, Flag-ISG15, Flag-BECN1, and Flag-ISG15-BECN1 and then infected with CSFV. **E** Expression of LC3 protein and fusion protein was probed by Western blot with an anti-LC3 antibody and an anti-Flag antibody, respectively. **F** The number of LC3 puncta per cell was determined by image analysis. **G** CSFV replication in these cells was quantified by RT-qPCR and IFA, respectively. Data (**B**, **C**, **F**, and **G**) represent the mean ± SD of three independent experiments and were measured in technical duplicate. Comparisons between groups were calculated using Student’s *t*-test. *P < 0.05; **P < 0.01; ***P < 0.001

To further understand how ISG15 influences the autophagic machinery during CSFV infection, BECN1 was overexpressed in ISG15-overexpressing cells. The LC3-II protein levels were evaluated in CSFV-infected cells that were co-transfected with CMV-ISG15, CMV and 3.1-BECN1, or 3.1. The LC3-II protein level and fluorescence signal were reduced by ISG15 but restored by BECN1 plasmid transfection, revealing that ISG15 regulates BECN1 to inhibit autophagy and CSFV replication (Figures 6A–C). To identify whether BECN1 is involved in ISGylation in PAMs during CSFV infection, BECN1 expression was evaluated in cells transfected with plasmids 3.1, 3.1-ISG15AA (expressing non-conjugative ISG15), or 3.1-ISG15GG (expressing wild-type ISG15) following CSFV infection. Interestingly, BECN1 expression in 3.1-ISG15AA-transfected cells was similar to that in 3.1-transfected cells, whereas some specific protein bands appeared only for cells transfected with 3.1-ISG15GG (Figure 6D). It has been proven that fusing ISG15 LRLRGG with the target protein can mimic the constitutively modified state of ISGylation [20–22]. BECN1 has been shown to have at least four alternative ISGylation sites at its N-terminus in H4 cells. Thus, we constructed Flag-ISG15-BECN1 recombinant plasmid in which LRLRGG of ISG15 was fused to the N-terminus of BECN1 to mimic BECN1 ISGylation. As shown in Figures 6E–G, LC3-II levels, the numbers of LC3 puncta, CSFV genomic RNA levels, and viral titers in Flag-ISG15-BECN1-expressing cells were obviously reduced compared with those in normal BECN1-expressing cells and PAMs transfected with 3.1 as a negative control. These results indicated that BECN1 ISGylated by ISG15 is unable to perform its function in autophagy and fails to promote CSFV replication.

### BECN1 interacts with the ISG15 E3 ligase HERC5

**Figure 7:**
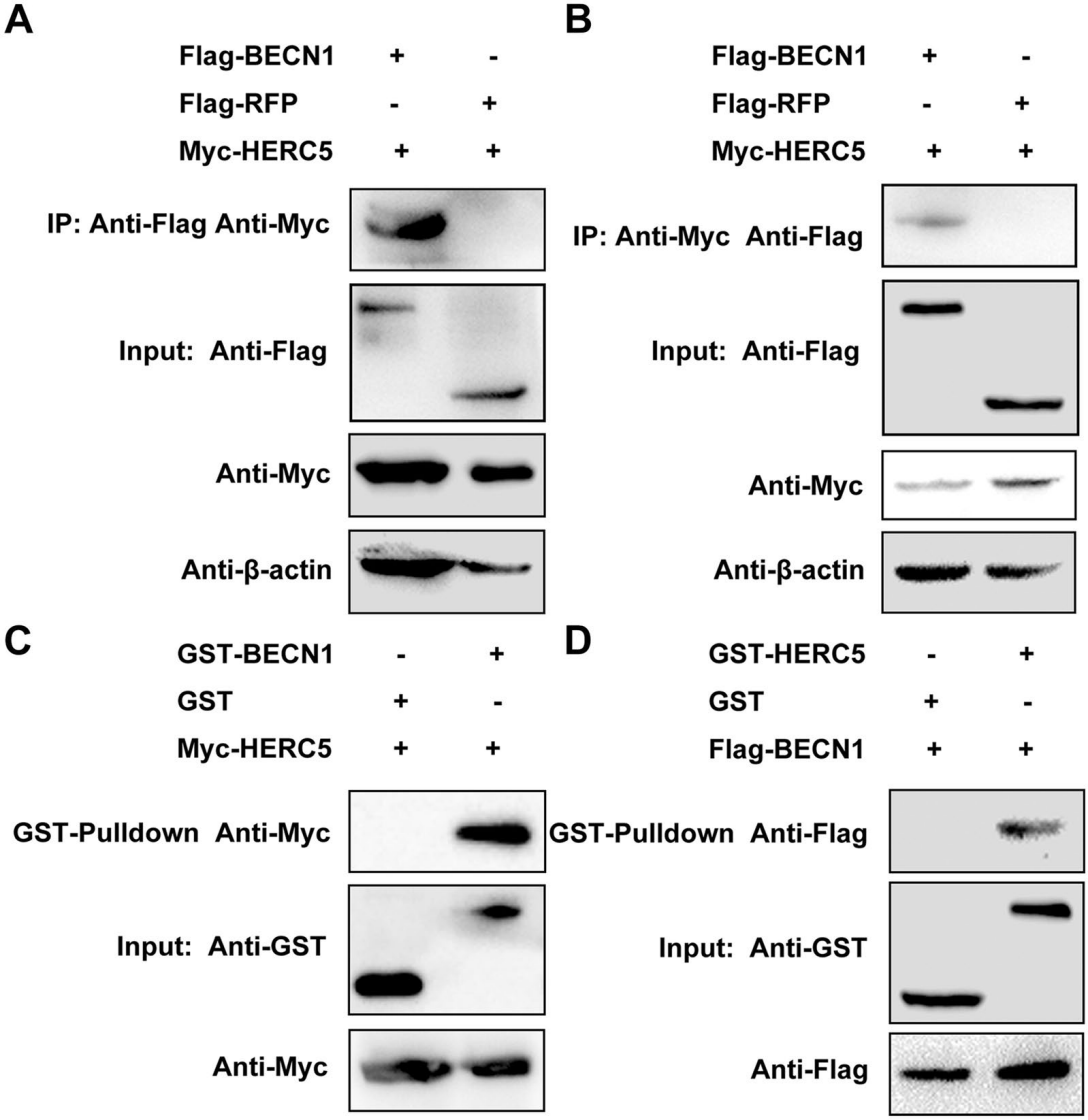
**BECN1 interacts with the ISG15 E3 ligase HERC5.A** PAMs were co-transfected with the plasmids Flag-BECN1 and Myc-HERC5 or Flag-RFP and Myc-HERC5 as a control. Cells were lysed and subjected to an IP assay with Anti-Flag M2 Affinity Gel. The precipitated proteins were detected by Western blot with an anti-Myc antibody. **B** PAMs were co-transfected with the plasmids Flag-BECN1 and Myc-HERC5 or Flag-RFP and Myc-HERC5 as a control. Cells were lysed and subjected to an IP assay with an anti-c-Myc immunoprecipitation kit. The precipitated proteins were analyzed by Western blot with an anti-Flag antibody. **C** GST and GST-BECN1 fusion proteins expressed in *E. coli* BL21 cells were conjugated with glutathione-Sepharose resin and incubated with lysate of HEK-293T cells overexpressing the Myc-HERC5 protein. The bound proteins were detected by Western blot with an anti-Myc antibody. **D** GST and GST-HERC5 fusion proteins expressed in *E. coli* were conjugated with glutathione-Sepharose resin and incubated with lysate of HEK-293T cells overexpressing the Flag-BECN1 protein. The bound proteins were detected by Western blot with an anti-Flag antibody

It is well known that ISG15 conjugation to protein substrates typically requires HERC5, which functions as an ISG15 E3 ligase to promote ISGylation [18]. Thus, we investigated whether a physical interaction exists between these proteins. Co-IP assays were performed, and the plasmids Flag-BECN1 and Flag-RFP were transfected into PAMs to detect Myc-tagged HERC5 protein. The result showed that Myc-HERC5 interacted with Flag-BECN1 but not Flag-RFP (Figure 7A). In the reciprocal co-IP assay, Flag-BECN1 was co-precipitated with Myc-HERC5, but the negative control Flag-RFP was not (Figure 7B). These findings demonstrated that BECN1 interacts with HERC5 in PAMs.

The GST pull-down assay was performed to further confirm the interaction between BECN1 and HERC5. GST-BECN1 and GST were expressed in *E. coli* BL21 cells, and Myc-HERC5 was expressed in HEK-293T cells. Western blot assays showed that Myc-HERC5 was captured by GST-BECN1 (Figure 7C). Furthermore, GST-HERC5 or GST was expressed in bacteria, and cell lysates containing Flag-BECN1 were analyzed. Flag-BECN1 was detected in GST-HERC5 complexes but not in GST complexes (Figure 7D). These results demonstrated that BECN1 interacts with HERC5 in vitro.

## Discussion

ISG15 has been proposed as an efficient host effector that defends against viral infections including that of human cytomegalovirus [17], human immune deficiency virus (HIV) [23], West Nile virus [24], and porcine reproductive and respiratory syncytial virus [25]. Macrophages are at the frontline of defense against pathogenic microorganisms. In our study, we provided the first strong evidence that ISG15 is an antiviral factor against CSFV in immune cells. Not only IFN-induced ISG15 but also overexpressed ISG15 alone obviously inhibited CSFV replication in PAMs (Figures 1 and 2). The expression of many ISGs is induced following type I IFN treatment. However, some of them exert independent antiviral functions that may affect CSFV replication [6, 7, 9]. This knowledge helps to explain why knockdown of ISG15 notably enhanced CSFV replication but did not totally neutralize the anti-CSFV activity of IFN-α (Figures 2D, E). In this study, *ISG15* was significantly increased during CSFV infection in PAMs. Similar experiments were performed in other cell types with consistent results. A recent study reported that IRF1 mediates the increase in ISG15 expression during CSFV infection in PK-15 cells. In this process, IRF1 interacts with nucleotides −487 to −325, which are located in the 5′ flanking region of the *ISG15* gene, following stimulation with dsRNA or CSFV [26]. Therefore, we speculated that ISG15 might be induced by CSFV in an IFN-independent manner in PAMs, which will be further verified in future studies. Similar results were obtained in research of Vaccine-C-strain; the ISG15 pathway was activated in response to Vaccine C-strain and ISG15 played a role in the rapid and early protection conferred by Vaccine C-strain [27]. In further research, we will test ISG15 effects on more CSFV genotypes to perfect the mechanisms of ISG15.

In addition to the unconjugated form of ISG15, which may be released from IFN-induced cells to the environment, the conjugated form of ISG15 can conjugate to numerous host and viral proteins [28]. With respect to other flaviviruses, overexpression of ISG15 significantly inhibits replication of Japanese encephalitis virus and West Nile virus via protein ISGylation [24, 29]. In this study, we used two methods to demonstrate that ISGylation was involved in the inhibition of CSFV replication mediated by ISG15. The C-terminal LRLRGG domain of ISG15 has been proven to be required for ISG15 conjugate formation [30, 31]. Firstly, we constructed a mutant of ISG15. Counteraction of ISGylation by mutation of the conjugation site (LRLRGG) to LRLRAA significantly increased CSFV replication, indicating that ISGylation plays an important role in limiting CSFV replication. The induced ISGylation was cleaved especially by USP18, which belongs to the family of deubiquitinating enzymes [32]. Secondly, we constructed a stable USP18-knockdown cell line. The ISGylation levels were increased and proliferation of CSFV was suppressed by downregulating USP18 expression, suggesting that inhibition of CSFV by ISG15 depends on ISGylation. Interestingly, knockdown of USP18 enhanced expression of ISG mRNA in this experiment. We found that knockdown of both ISG15 and USP18 upregulated ISG expression and exerted opposite effects on CSFV. In ISG15-knockdown cells pretreated with IFN-α, the replication of CSFV was upregulated, even though ISG expression was upregulated. With regard to CSFV inhibition, the effect of ISGylation of ISG15 was predominant compared with the effect of other ISGs.

The E2 and NS5A proteins of CSFV are colocalized with autophagosome-like vesicles; autophagy provides vesicle membranes to promote CSFV replication and maturity in PK-15 cells and PAMs [14, 33]. Our research showed that ISG15 has a negative effect on both CSFV-induced and rapamycin-induced autophagy. When we inhibited autophagy levels, we found that downregulated ISG15 expression did not promote CSFV replication.

BECN1 is a key initiator of autophagy and associates with PtdIns3K, which mediates biogenesis and the dynamics of subcellular membranes and is involved in the autophagic process [34, 35]. In H4 cells and HepG2 cells, BECN1 has been identified as a target protein of ISGylation. In addition, ISGylation of BECN1 inhibits PI3KC3 complex activation, which plays a pivotal role in autophagy, and USP18 positively regulates autophagy by promoting de-ISGylation of BECN1 [36, 37]. Nonetheless, in swine cells, the relationship between ISG15 and autophagy has not been reported. We showed that BECN1 was ISGylated during the antiviral action of ISG15 against CSFV. This modification affected the autophagy pathway, leading to the inhibition of CSFV replication. We further validated this result using fusion Flag-ISG15-BECN1 protein. Such proteins have been confirmed to mimic the ISG15-modified state of a protein and are especially useful when the modification site is near the terminus of the protein [18, 21, 22]. Flag-ISG15-BECN1 expression was not able to initiate autophagy and induce CSFV replication like BECN1. These results are also consistent with our hypothesis that modification of autophagy by ISGylation of BECN1 impedes CSFV utilization of autophagy for self-production.

HERC5 functions as an ISG15 E3 ligase to promote ISGylation [18]. Recent studies support the contention that ISGylation of target proteins is regulated by HERC5. HERC5 especially catalyzes the ISGylation of ISG15 onto IRF3, which regulates innate antiviral activity in Sendai virus infection [38]. ISGylation of the target protein Parkin is specifically mediated by the ISG15 E3 ligase HERC5 [39]. As shown in Figure 7, HERC5 appeared to target BECN1, indicating that HERC5 may mediate covalent ISG15 conjugation to BECN1 in PAMs. ISG15 may also regulate autophagy-independent activities whose contributions to CSFV proliferation remain to be investigated. However, our current findings highlight the functional importance of ISG15 with regard to the antiviral activity of IFN and confirm that ISG15 functions as an anti-CSFV effector by regulating autophagy.

## Data Availability

All data generated or analyzed during this study are included in this published article.

## Abbreviations

3MA: 3-methyladenine
BECN1: beclin-1
Co-IP: co-immunoprecipitation
CSF: classical swine fever
CSFV: classical swine fever virus
DMEM: Dulbecco’s minimal essential medium
FBS: fetal bovine serum
FITC: fluorescein isothiocyanate
GST: glutathione S-transferase
HERC5: HECT and RLD domain containing E3 ubiquitin protein ligase 5
hpi: hour post-infection
IFA: indirect immunofluorescence assay
IFNs: interferons
ISGs: interferon-stimulated genes
MAb: monoclonal antibody
MOI: multiplicity of infection
PAb: polyclonal antibody
PAMs: porcine alveolar macrophages
PBS: phosphate-buffered saline
PCR: polymerase chain reaction
RT-qPCR: real-time quantitative polymerase chain reaction
SD: standard deviation
SDS-PAGE: sodium dodecyl sulfate-polyacrylamide gel electrophoresis
shRNA: short hairpin RNA
shN: scrambled control shRNA
TBST: Tris-buffered saline containing 0.5% Tween 20
TCID_50_: 50% tissue culture infectious dose
TEM: transmission electron microscopy
USP18: ubiquitin-specific protease 18
